# Changing pattern of drowning in Chaharmahal and Bakhtiari province in central of Iran during 2018-2020 

**Published:** 2022-01

**Authors:** Salman Yadollahi, Sadegh Heydarpoor Dastgerdi, Kamran Mohammadi Janbazloufar

**Affiliations:** ^*a*^ Pre-Hospital Emergency Research Center, Shahrekord University of Medical Sciences, Shahrekord, Iran.; ^*b*^ PhD Student, Department of Health in Disasters and Emergencies, Isfahan University of Medical Sciences, Isfahan, Iran.

## Abstract

**Background:**

Drowning is regarded as a danger to people’s health especially in the summer season considering the geographical location of Chaharmahal and Bakhtiari province. This study aimed to compare the drowning cases assisted by the Emergency Medical Services (EMS) of the province during the first six months of 2018 with similar periods in 2019 and 2020.

**Methods:**

In a cross-sectional study, based on the data obtained from the Shahrekord's pre-hospital emergency records, the reports of 103 drowning cases who were rescued by Chaharmahal and Bakhtiari’s EMS in the first 6 months of 2018-2020 were reviewed. Variables of age and sex of the drowned, patient’s status (death, hospitalization), place of drowning, time of occurrence, mission response time, and month of the incident were provided to the authors by the Quality Control Team. These data were entered into the SPSS16 and analyzed by t-test and chi-square tests. The level of significance was considered 0.05.

**Results:**

The trend of drowning cases in the first six months of 2020 increased by 115% compared with that of 2018. The results indicated that of 103 drownings, 82 (79.62%) cases were male and 21 (20.38%) were female. The highest number of drownings in terms of age group was 21 (20.38%) cases in 15- to 19-year-old ones. Most of the drowning places were rivers with 83 cases (80.58%) followed by dams with 7 cases (6.79%). Out of the total number of drowning cases, 83 cases (87.1%) were transferred to the hospital and 20 cases (20.38%) died.

**Conclusions:**

It seems that most of the places prone to drowning are the rivers of Chaharmahal and Bakhtiari. Therefore, more efforts should be made to educate the public about the dangers of swimming in rivers with unsafe beds and unknown conditions.

**Keywords:**

Drowning, Emergency Medical Services, Chaharmahal and Bakhtiari, Iran

